# New reference genome assembly for the declining American Bumble Bee, *Bombus pensylvanicus*

**DOI:** 10.1093/g3journal/jkaf181

**Published:** 2025-08-11

**Authors:** Jeffrey D Lozier, Rena M Schweizer, Sheina B Sim, Jonathan Berenguer Uhuad Koch, Michael G Branstetter, Ligia R Benavides, Scott M Geib, Jay D Evans

**Affiliations:** Department of Biological Sciences, The University of Alabama, Tuscaloosa, AL 35487, United States; U.S. Department of Agriculture, Agricultural Research Service, Pollinating Insects Biology, Management, Systematics Research Unit, Logan, UT 84341, United States; Division of Biological Sciences, University of Montana, Missoula, MT 59812, United States; U.S. Department of Agriculture, Agricultural Research Service, Daniel K. Inouye U.S. Pacific Basin Agricultural Research Center, Tropical Pest Genetics and Molecular Biology Research Unit, Hilo, HI 96720, United States; U.S. Department of Agriculture, Agricultural Research Service, Pollinating Insects Biology, Management, Systematics Research Unit, Logan, UT 84341, United States; Pacific Cooperative Studies Unit, College of Natural Sciences, University of Hawai‘i at Mānoa, Honolulu, HI 96822-2279, United States; U.S. Department of Agriculture, Agricultural Research Service, Pollinating Insects Biology, Management, Systematics Research Unit, Logan, UT 84341, United States; U.S. Department of Agriculture, Agricultural Research Service, Pollinating Insects Biology, Management, Systematics Research Unit, Logan, UT 84341, United States; Museum of Comparative Zoology, Harvard University, 26 Oxford Street, Cambridge, MA 02138, United States; U.S. Department of Agriculture, Agricultural Research Service, Daniel K. Inouye U.S. Pacific Basin Agricultural Research Center, Tropical Pest Genetics and Molecular Biology Research Unit, Hilo, HI 96720, United States; Bee Research Laboratory, Agricultural Research Service, United States Department of Agriculture, Beltsville, MD 20705, United States

**Keywords:** bumble bees, pollinator, biodiversity, conservation, bees, genome assembly

## Abstract

We present the first chromosome-level genome assembly for *Bombus pensylvanicus*, a historically widespread native pollinator species that was distributed across eastern North America but has subsequently undergone declines in range area and local relative abundance. This species has been of significant interest as a model for understanding both patterns and possible causes of bumble bee decline in the region, including the role of genetic variation. Here we present a chromosome-level reference genome assembled using Pacific Biosciences singe-molecule HiFi sequences and Hi-C data and annotated using evidence derived from RNA sequencing of multiple tissue types. The *B. pensylvanicus* genome has a total length of ∼352.6 Mb and was assembled into a total of 224 scaffolds, with 19 primary pseudomolecules representing putative chromosomes and an N50 = 14.872 Mb. Annotation with the Eukaryotic Genome Annotation Pipeline—External (EGAPx) identified 11,411 genes (10,263 protein coding), and BUSCO analysis of 5,991 Hymenoptera-specific BUSCO groups indicated a completeness for the proteins of 99.0% (98.6% single-copy, 0.5% duplicated) and for the genome of 98.5% (98.2% single-copy, 0.3% duplicated). We present synteny analyses with other recently assembled *Bombus* genomes representing different subgenera and examine the distribution of repetitive regions of the genome relative to the distribution of genes and noncoding RNAs.

## Introduction

Bumble bees (Hymenoptera: Apidae, *Bombus* Latreille, 1802) are important native pollinators of agricultural crop plants and wildflowers (Thorp et al. 2002; Velthuis and van Doorn 2006; Goulson 2010). Unfortunately, once widespread and common species have declined over the last several decades, with many species showing evidence of reductions in both geographic range and local abundance (Williams and Osborne 2009; Cameron and Sadd 2020). Evolutionary aspects of bumble bee biology, such as genetic diversity and gene flow to genome-scale analyses of molecular evolution and natural selection, have become a common component of bumble bee conservation studies (Lozier and Zayed 2017; Sun et al. 2021; Liu et al. 2024; Mola et al. 2024), and increasing availability of species-specific reference genomes across the genus is poised to greatly accelerate such analyses (Sadd et al. 2015; Heraghty et al. 2020; Sun et al. 2021; Koch et al. 2024; Martínez et al. 2024; Toth et al. 2024).

*Bombus* (subgenus *Thoracobombus*) *pensylvanicus* (De Geer, 1773), “the American Bumblebee,” was once wide ranging across eastern North America, primarily in the central and eastern United States, southern Canada and Mexico. The species has declined, especially in northern and easternmost parts of its historic range (Colla and Packer 2008; Grixti et al. 2009; Cameron et al. 2011) and is currently classified as vulnerable by the International Union for Conservation of Nature (IUCN) (Hatfield et al. 2015a). Pinpointing causes of species decline is difficult (reviewed in Hatfield et al. 2015a), but hypotheses include greater prevalence of the pathogen *Nosema bombi* (Cameron et al. 2011), modification of open field and grassland habitats (Grixti et al. 2009), and possible population genetic factors (Lozier et al. 2011).

*Bombus pensylvanicus* has been the target of several genetic studies, including examination of population structure and taxonomic status (Lozier et al. 2011; Beckham et al. 2024) and comparisons of genetic diversity to other codistributed but seemingly stable *Bombus* species (Lozier and Cameron 2009 ; Cameron et al. 2011; Lozier 2014). However, some conflicting results from such studies may be resolved with better genomic resources. For example, microsatellites suggested that *B. pensylvanicus* had reduced heterozygosity compared with more stable *B. impatiens* and *B. bimaculatus* across the eastern USA (Cameron et al. 2011). Conversely, genome-wide restriction site-associated DNA sequencing markers suggested that range-wide estimates of nucleotide diversity may be more similar between *B. pensylvanicus* and *B. impatiens* (Lozier 2014). Whole genome resequencing-based analyses might aid in better understanding such discrepancies, while at the same time enabling comparisons of genome structure and genetic features that explain different demographic trajectories among species. Recently, high-quality genomes for other North American *Bombus* species have been published with improved methods that facilitate chromosome-scale assembly, including widespread species such as *B. impatiens* (Toth et al. 2024) and other declining species such as *B. affinis* (Koch et al. 2023). Genome resequencing-based population genetic comparisons of diversity among stable and declining species would thus benefit from a reference genome for *B. penyslvanicus*, and a reference genome will be valuable for other studies including analyses of natural selection (e.g. Heraghty et al. 2022) and other traits related to conservation, such as genetic factors associated with susceptibility to parasites.

We report a new reference genome for *B. pensylvanicus* that contains chromosome-scale scaffolds assembled using long-read Pacific Biosystems (PacBio) HiFi and Element Aviti Hi-C sequencing as part of the Beenome100 project (https://www.beenome100.org/), a United States Department of Agriculture-led initiative to assemble and annotate genomes from more than 100 native U.S. bee species. We employ a new external version of the National Center for Biotechnology Information (NCBI) Eukaryotic Genome Annotation Pipeline (Thibaud-Nissen et al. 2016; https://github.com/ncbi/egapx) to generate gene structural and functional annotations. We also analyze the distribution of repetitive elements across the genome and synteny to other *Bombus* subgenera. The *B. pensylvanicus* genome adds to the growing number for North American bumble bees and represents the first annotated assembly for North American members of the subgenus *Thoracobombus* to date. This genome will provide a valuable resource for evolutionary studies in this charismatic but threatened native pollinator species.

## Methods

### Samples used for sequencing

A female *Bombus pensylvanicus* worker (JDL3197) was collected in Tuscaloosa, AL (33.1925N, 87.5319W) on 29 August 2022. Sample information is available at NCBI [BioSample: SAMN40264069; SAMN47609296; SAMN47609294; SAMN47609295; Sample name: JDL3197; BioProject PRJNA1083979 (principal); PRJNA1083978 (alternate); Assembly: JBBAXX000000000 (principal); JBBAXY000000000 (alternate)]. The live specimen was snap frozen in liquid nitrogen and subsequent storage at −80 °C until transport on dry ice to the United States Department of Agriculture-Agricultural Research Service Pacific Basin Agricultural Research Center in Hilo, Hawaii, USA.

### HiFi, Hi-C, and RNA sequencing

Sequencing methods largely follow those used for the recent assembly of *Bombus huntii* (Koch et al. 2024) with small modifications. Genomic DNA was isolated from a slice of abdominal tissue using the Qiagen MagAttract HMW DNA Kit (Qiagen, Hilden Germany) fresh or frozen tissue protocol and purified using 2:1 polyethylene glycol with solid-phase reversible immobilization beads (DeAngelis et al. 1995). DNA was quantified using the dsDNA BR Qubit assay (Thermo Fisher Scientific, Waltham, Massachusetts, USA) with the fluorometry function of a DS-11 Spectrophotometer and Fluorometer (DeNovix Inc, Wilmington, Delaware, USA). Purity was then determined using OD 260/230 and 260/280 ratios from the UV–Vis spectrometer feature of the DS-11. DNA was sheared to a mean size of 15–20 kb with a Diagenode Megaruptor 2 (Denville, New Jersey, USA) for generating a SMRTbell library using the SMRTbell prep kit 3.0, using the manufacturers protocols for low input samples (Pacific Biosciences, Menlo Park, California, USA). Ampure PB beads (Pacific Biosciences) were used to remove fragments <3 kb in length from the library. The resulting PacBio SMRTbell library was then quantified using Qubit HS dsDNA as above and sized on an Agilent Fragment Analyzer (Agilent Technologies, Santa Clara, California, USA) using a high sensitivity large fragment kit to determine molar concentration. The prepared library was bound for sequencing and sequenced on a single 8 M SMRT cell on a PacBio Sequel IIe instrument using default parameters, outputting HiFi reads for subsequent analysis.

Hi-C libraries were generated from cross-linked tissue from the same specimen using an Arima Hi-C kit (Arima Genomics, San Diego, California, USA), following the Arima low input protocol using restriction enzymes *Dde*I and *Dpn*II. After the proximity ligation step, DNA was sheared using a Diagenode Bioruptor (Denville, New Jersey, USA). A Swift Accel NGS 2S Plus kit (Integrated DNA Technologies, Coralville, Iowa, USA) was used to prepare Illumina sequencing libraries with insert size range of 200–600 bp and 150-bp paired-end sequencing was performed on an AVITI sequencer (Element Biosciences, San Diego, California, USA).

Three separate RNA sequencing (RNA-seq) libraries were generated from head, abdomen, and thorax tissue samples. Total RNA was extracted using Direct-zol-96 MagBead RNA kit (Zymo) on a Kingfisher Flex 96 system (Thermo Scientific), and strand-specific poly(A) libraries produced using the NEBNext Ultra II Directional RNA Library Prep Kit and the poly(A) mRNA Magnetic Isolation Module (New England Biolabs, Ipswich, MA). 150 bp paired-end sequencing was conducted on a partial lane on an AVITI sequencer as above.

### Genome assembly, scaffolding, and quality control

We filtered adapters from HiFi reads using HiFiAdapterFilt v0.2.3 (Sim et al. 2022) with default parameters. We performed genome assembly using HiFiASM v0.16.1-r375 (Cheng et al. 2021). This preliminary contig assembly was assessed for quality and completeness using BlobToolKit v 2.6.1 (Kumar et al. 2013; Laetsch and Blaxter 2017; Challis et al. 2020) and the Benchmark of Single-Copy Orthologs (BUSCOs) (Waterhouse et al. 2018; Manni et al. 2021). We used GenomeScope2 (Ranallo-Benavidez et al. 2020) to estimate genome size and coverage, specifying a ploidy of 2, and assessed kmer frequencies with Merqury (Rhie et al. 2020). We purged duplicates using purge_dups v1.2.5 (Guan et al. 2020) to increase the accuracy of the principal and alternate haplotypes by separating duplicate regions that result from diploidy into each haplotype. With the HiC data, we scaffolded the contigs using Yet Another Hi-C Scaffolding tool (Zhou et al. 2023), which also generates HiC contact maps via the Juicebox (Dudchenko et al. 2018) tool set. We manually curated the scaffolded assembly in Juicebox and converted the curated scaffold using the Juicebox “juicebox_assembly_converter.py” script. We re-ran Blobtools to assess assembly quality and identify taxonomic origin of scaffolds by assigning scaffolds to their closest taxon using BLAST (Altschul et al. 1997) and Diamond (Buchfink et al. 2015) searches to NCBI nt and UniProt databases, respectively, within BlobToolKit. We retained scaffolds that were classified as “arthropod” and “no-hit” (excluded scaffolds available at doi:10.6084/m9.figshare.28661291). The no-hit scaffolds appeared to arise from the BLAST step timing out, especially for large scaffolds, possibly due to large amounts of repetitive DNA (see Results). We manually checked random sequences across the length of larger “no-hit” scaffolds using BLASTn and based on top hits to *Bombu*s species they were all retained in the final assembly. These scaffolds were also confirmed as *Bombus-*derived by the presence of Hymenopteran BUSCOs and synteny analyses (see below). Following the MitoHiFi v2 workflow (Uliano-Silva et al. 2023), we identified the mitochondrial genome, specifying *Bombus longipennis* (NCBI accession: NC_057952.1) as the closest reference genome, and used Mitos (Bernt et al. 2013) within MitoHiFi to identify and remove the contig associated with the mitochondrial genome (available at doi:10.6084/m9.figshare.28661291). Retained scaffolds were assigned to putative chromosomes after ordering by size and ensuring that all designated scaffolds contained Hymenopteran BUSCOs.

### Genome annotation and synteny analysis

Genome annotations were performed using the external version of NCBI Eukaryotic Genome Annotation Pipeline (Thibaud-Nissen et al. 2016) (EGAPx 0.3.1-alpha; https://github.com/ncbi/egapx) to generate annotations (gene, mRNA, CDS, ncRNA). Evidence for annotations used the provided Hymenoptera protein set (Taxid 7399) and the paired-end *B. pensylvanicus* RNA-seq libraries for head, thorax, and abdomen described above. The annotation process using EGAPx otherwise used default settings as defined in run_params.yaml file (available at doi:10.6084/m9.figshare.28661291). After annotation, we used BUSCO v5.8.2 (Manni et al. 2021) with the Hymenoptera dataset (hymenoptera_obd10) containing 5,991 BUSCOs to determine completeness of the assembled genome (-m genome) and the annotated protein set (-m protein), only considering the single longest isoform per annotated gene model (available at doi:10.6084/m9.figshare.28661564).

We identified repetitive elements with RepeatModeler v2.0.6 (Flynn et al. 2020) and RepeatMasker 4.1.7 (Smit et al. 20132015), installed using the Dfam TETools container v1.9 (https://github.com/Dfam-consortium/TETools) with RECON v1.08 (Bao and Eddy 2002), RepeatScout v1.0.7 (Price et al. 2005), TRF v4.09.1 (Benson 1999), RMBlast v2.14.1, UCSC genome browser utilities v413 (Perez et al. 2025), LTRharvest v1.6.4 (Ellinghaus et al. 2008), MAFFT v7.7471 (Katoh et al. 2002), cd-hit v4.8.1 (Li and Godzik 2006), HMMER v3.4 (Eddy 2023), NINJA (Wheeler 2009 ), and LTR_retriever v 2.9.0 (Ou and Jiang 2018). The RepeatModeler pipeline was used for de novo transposable element detection in the *B. pensylvanicus* assembly and then combined with the Dfam 3.8 database partition 7 (dfam38-1_full.7.h5) (Storer et al. 2021) for the family Apidae to create a custom species-specific RepeatMasker library (available at doi:10.6084/m9.figshare.28661591). Protein coding genes and noncoding RNA (ncRNAs) from EGAPx annotations, repeat elements identified by RepeatMasker, and GC% summaries by BEDtools v2.31.1 were plotted using the R version 4.4.1 (R Core Team 2024) package CIRCLIZE (Gu et al. 2014).

We performed synteny analysis to visually evaluate that putative chromosomes were mostly homologous to other bumble bee genomes, examine genome rearrangements between several common subgenera, and examine the origins of a 19th putative chromosome in *B. pensylvanicus* (see Results; many *Bombus* have 18 chromosomes). We evaluated synteny between the *B. pensylvanicus* genome and recent chromosome-scale RefSeq-annotated assemblies for two additional North American bumble bees representing the subgenera *Pyrobombus* (*B. huntii*) (Koch et al. 2024) and *Bombus sensu stricto* (*B. affinis*) (Koch et al. 2023) subgenera. We obtained the translated CDS protein FASTA and GFF files for *B. huntii* from RefSeq GCF_024542735.1 and for *B. affinis* from RefSeq GCF_024516045.1. We used GENESPACE v1.4 (Lovell et al. 2022) to produce synteny plots (available at doi:10.6084/m9.figshare.28661705). For each genome, structural annotations were converted from the GFF file into BED formatted coordinates using the parse_annotations function. The GENESPACE pipeline uses OrthoFinder 3.0.1b1 (Emms and Kelly 2015; Emms and Kelly 2019) to assign orthologous groups among the annotated species. To visualize the variation in chromosome structure, GENESPACE riverine plots were used to map syntenic blocks and rearrangements (e.g. gaps, inversions, translocations) among the genomes. Datasets and scripts are available on Figshare (Lozier 2025).

## Results and discussion

**Fig. 1.**
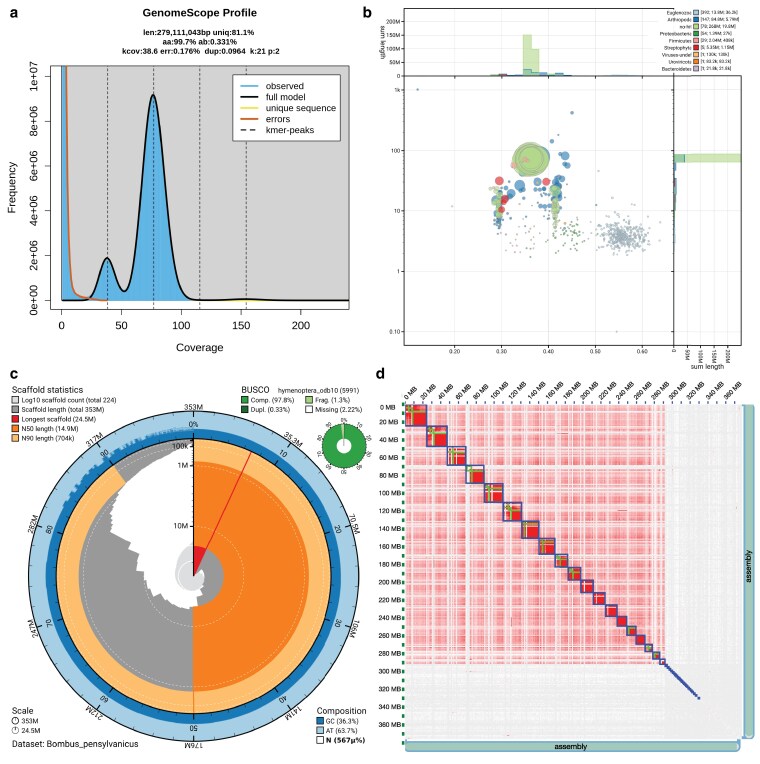
Genome assembly details for *Bombus pensylvanicus*. a) Genomescope2 plot demonstrating high kmer coverage and low error rate, assuming a diploid genome. b) Blob plot for scaffolded assembly prior to removing nonarthropod contaminants. c) Snail plot showing quality metrics for final, clean scaffolded assembly; note that the BUSCO scores reflect the report by BlobToolKit and differ slightly from values in the main text and Table 1 reported from analyses of the final assembly FASTA and protein set using the standalone BUSCO v5.8.2 software. d) Juicebox Hi-C contact heatmap after manual curation showing scaffolding of contigs into 19 chromosomes (largest bold-outlined boxes) and the unplaced scaffolds. Shading intensity indicates interaction frequency between regions of DNA that are physically close to one another. The *x*- and *y*-axis labels show the approximate size of the assembly in megabases (MB).

The PacBio HiFi sequencing produced sufficient data for a high-quality assembly, with 2,026,087 HiFi reads and 22.8 Gb of data. Sequencing of the Hi-C library generated 53,890,722 read pairs and RNA sequencing generated 26,162,923 read pairs for head, 16,107,971 for thorax, and 40,680,345 for abdomen for use in gene annotation. BlobTools results from the initial assembly revealed a substantial fraction of nonArthropod scaffolds, with 483 taxonomic matches to protists (*n* = 392, Euglenozoa), Proteobacteria (*n* = 54), Firmicutes (*n* = 29, bacteria), plants (*n* = 5, Streptophyta), undefined viruses (*n* = 1), Uroviricota (*n* = 1, bacteriophage viruses), and Bacteroidetes (*n* = 1), accounting for around 22.8 Mb of sequence. We retained scaffolds assigned to both arthropod (*n* = 147) and “no-hit” (*n* = 78). The total estimated sequencing coverage of PacBio reads for these scaffolds was ∼75× (Fig. 1; Supplementary Fig. 2). The final *B. pensylvanicus* assembly (principal assembly JBBAXX000000000; alternate assembly JBBAXY000000000) was 352.572 Mb in length across 244 contigs, with contig N50 = 10.03 Mb, and 224 scaffolds, with a scaffold N50 = 14.872 Mb and 36.33% GC content. Hi-C scaffolding indicated 19 major scaffolds corresponding to putative chromosomes (length of top 19 scaffolds = 292.156, ranging in size from 5.792 Mb to 24.541 Mb) (Fig. 1, Table 1, Supplementary Fig. 1). One scaffold (scaffold0168) was identified as the putative mitochondrial genome.

**Table 1.** Feature count summary of the *B. pensylvanicus* assembly and annotation using the NCBI EGAPx annotation pipeline.

	Feature count
**Genome summary**	
Putative chromosomes	19
Scaffolds	244
Size (Mb)	352.572
N50 (Mb)	14.872
GC %	36.33
**BUSCO (genome/protein)**	
Complete	98.5%/99.0%
Complete single-copy	98.2%/98.6%
Complete duplicated	0.3%/0.5%
Fragmented	0.7%/0.2%
Missing	0.9%/0.8%
**Genes**	11,411
Nontranscribed Pseudogene	103
Protein coding	10,263
Noncoding	1,045
Genes (has variants)	3,582
Genes (partial)	23
Genes (major correction)	174
Genes (premature stop)	52
Genes (has frameshifts)	153
**mRNAs**	18,001
mRNAs (exon ≤ 3nt)	7
mRNAs (partial)	23
mRNAs (correction)	153
**Noncoding RNAs**	1,156
**CDSs**	18,001
CDSs (exon ≤ 3nt)	308
CDSs (partial)	23
CDSs (correction)	153
CDSs (major correction)	174
CDSs (premature stop)	52
CDSs (has frameshifts)	153

BUSCO v5.8.2 results presented for the genome/protein data sets using the Hymenotera odb10 data set with 5,991 BUSCOs.

Annotation using the EGAPx pipeline (Table 1) predicted 11,411 genes and pseudogenes (10,263 protein coding genes), 18,001 mRNAs, and 1,045 noncoding RNAs (ncRNAs) which is consistent to other contemporary bumble bee genome annotations performed by NCBI for RefSeq, except for somewhat lower numbers for ncRNAs and CDSs (see Koch et al. 2024). Most annotations (99.2%) were contained within the 19 putative chromosomes. BUSCO analysis (v5.7.1) using the hymenoptera_odb10 data set indicated a complete genome, with 98.5% of 5,991 benchmarking single copy orthologs detected (98.2% single copy, 0.3% duplicated, 0.7% fragmented, 0.9% missing) for the genome sequence and 99% detected (98.6% single copy, 0.5% duplicated, 0.2% fragmented, 0.8% missing) for the annotated protein set. All 19 chromosomal scaffolds contained hymentoperan BUSCOs and no unplaced scaffolds contained BUSCOs.

**Fig. 2.**
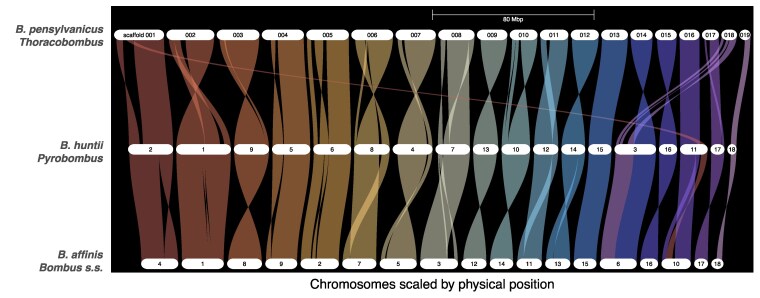
A GENESPACE riparian plot synteny map (top to bottom) of *B. pensylvanicus* (*Thoracobombus* subgenus), B*. huntii* (*Pyrobombus* subgenus) and *B. affinis* (*Bombus sensu stricto* subgenus). The plot is organized according the 19 *B. pensylvanicus* pseodochromosomes (largest to smallest in physical size), with colored braids representing syntenic blocks between chromosomes of the other bee genomes.

OrthoFinder identified and aligned 9,871 orthogroups between *B. pensylvanicus*, *B. huntii*, and *B. affinis* for synteny analysis using GENESPACE (Fig. 2). Overall synteny was high, with most changes being small within-chromosome rearrangements, with the only major rearrangement being the origin of the putative *B. pensylvanicus* chromosome scaffold0018 as orthologous to the terminus of a longer chromosome in the other species (chromosome 3 and 6 in *B. huntii* and *B. affinis*, respectively; Fig. 2). Based on prior karyotyping work (Owen et al. 1995), we had anticipated that *B. pensylvanicus* would have 18 chromosomes, the most common haploid number in *Bombus*. However, while some *Thoracobombus* have 18 chromosomes (*B. dahlbomii*; Martínez et al. 2024) other closely-related North American members of the subgenus *Thoracobombus* (e.g. *B. californicus*) have a haploid number of 19 (Owen et al. 1995), and the chromosome number in this subgenus generally appears variable (e.g. the European species *B. pascuorum* has 17 chromosomes; Crowley et al. 2023). The homology of the 19th *B. pensylvanicus* chromosome with approximately one-third to one-half of a larger chromosome in other subgenera indicates an origin from a single large-effect rearrangement, however, because of the large amounts of repetitive DNA near the ends of chromosomes in *B. pensylvanicus* it is also possible that this chromosome arises due to a misassembly. Additional assemblies of close *B. pensylvanicus Thoracobombus* relatives may help resolve this issue.

**Fig. 3.**
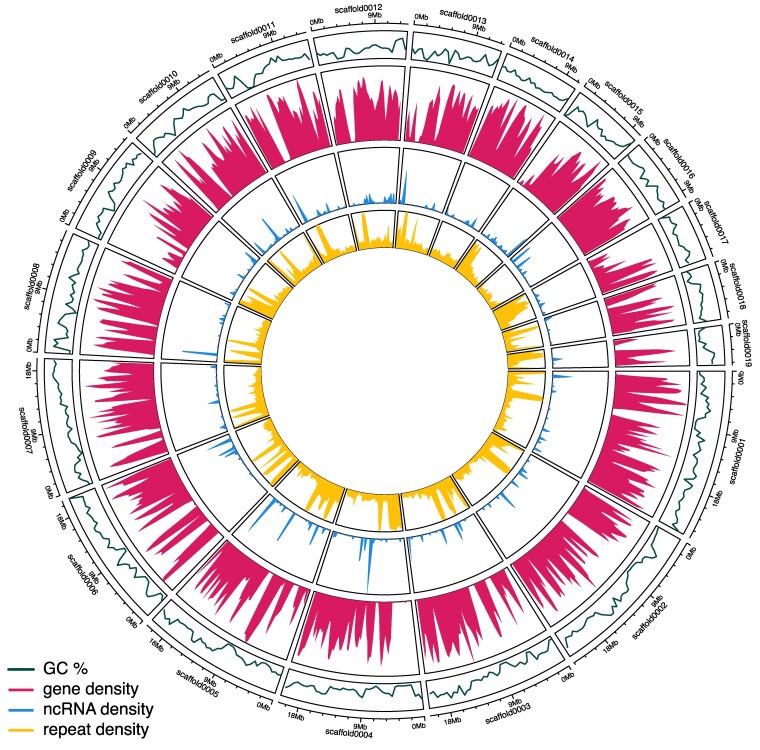
Summary features of the main 19 identified chromosomes for *B. pensylvanicus*. In order from outer to inner, the rings show scaffold identifiers and size, followed by GC%, gene density, ncRNA density, and repeat density. Statistics are summarized over 500 Mb windows.

The *B. pensylvanicus* genome's repetitive content was 46.77% as determined by RepeatMasker using a *de novo B. pensylvanicus* RepeatModeler library merged with the Dfam 3.8 Apidae repeat families (Table 2). 13.91% of the genome was classified as retroelements, including 3.11% Long Interspersed Nuclear Elements (LINEs) and 10.79% Long Tandem Repeat (LTR) elements (mostly Gypsy family), 16.39% was classified as DNA transposons, and 13% was unclassified repeats. The distribution of repetitive elements across the scaffolds was inversely related to gene density, with repetitive regions clustering into large gene-free regions of the chromosomes (Fig. 3). These gene-free regions were also associated with notable reductions in GC% (Fig. 2). Much of the repetitive DNA across the genome could be attributed to the small unplaced scaffolds, which were mostly composed of repetitive DNA and as noted above, very few genes (Supplementary Fig. 3). However, running RepeatMasker on the 19 assembled chromosomes indicated that main chromosomes were also repetitive at 36% repetitive content (9.17% retroelements, 13.05% DNA transposons, 11.8% unclassified) (Supplementary File 1).

**Table 2.** Summary of repeat elements in the full *B. pensylvanicus* assembly from RepeatMasker with a custom library including a *de novo* RepeatModeler library for *B. pensylvanicus* combined with models for Apidae from the Dfam 3.8 FamDB partition 7 (taxon 7458).

Element category	*N*	Length (bp)	% of genome
**Retroelements**	54,148	49,050,147	13.91%
SINEs	151	17,464	0.00%
Penelope	880	84,163	0.02%
LINEs	20,189	10,977,876	3.11%
CRE/SLACS	0	0	0
L2/CR1/Rex	1,216	330,426	0.09%
R1/LOA/Jockey	9,433	6,576,557	1.87%
R2/R4/NeSL	593	418,970	0.12%
RTE/Bov-B	733	219,427	0.06%
L1/CIN4	266	28,448	0.01%
LTR elements	33,808	38,054,807	10.79%
BEL/Pao	3,127	2,011,872	0.57%
Ty1/Copia	2,901	898,286	0.25%
Gypsy/DIRS1	25,295	34,261,284	9.72%
Retroviral	606	67,410	0.02%
**DNA transposons**	74,505	57,783,368	16.39%
Hobo-Activator	6,727	755,554	0.21%
Tc1-IS630-Pogo	37,676	8,030,576	2.28%
En-Spm	0	0	0.00%
MULE-MuDR	11,831	44,306,248	12.57%
PiggyBac	8,399	2,159,844	0.61%
Tourist/Harbinger	530	81,402	0.02%
Other	211	40,552	0.01%
**Rolling-circles**	2,033	409,074	0.12%
**Unclassified**	207,593	45,850,272	13.00%
**Total interspersed repeats**		152,767,950	43.33%
**Small RNA**	1,809	5,955,068	1.69%
**Satellites**	449	64,673	0.02%
**Simple repeats**	79,702	4,895,875	1.39%
**Low complexity**	16,101	831,331	0.24%

Because of the seeming impact of repeats on taxonomic identification of scaffolds during the BlobToolKit quality control step (see above), we were interested in examining if *B. pensylvanicus* had an unusually large amount of repetitive DNA compared with other recent *Bombus* genome assemblies derived from similar sequencing data and assembly approaches. We examined repetitive DNA using the same methods as described for *B. pensylvanicus* above (species-specific RepeatModeler libraries + Apidae Dfam) and found that *B. pensylvanicus* did have the greatest proportion of repetitive elements in the main assembled chromosomes (36%), but was not hugely different (∼3 to 8% greater), with 33.5% repetitive DNA (7.2% retroelements, 10.4% DNA transposons, 10.6% unclassified) in *B. huntii* (Koch et al. 2024), 27.8% (7.3% retroelements, 6.3% DNA transposons, 11.9% unclassified) in *B. affinis* (Koch et al. 2023), and 28.7% (9.17% retroelements, 5.4% DNA transposons, 11.9% unclassified) in *B. impatiens* (Toth et al. 2024) assembles (Supplementary File 1). It is important to note that the exact assignment of repeats to family is dependent on the library used, and that the custom libraries contain both curated and uncurated families from Dfam 3.8. Restricting analyses to species-specific RepeatModeler libraries without Dfam detected somewhat smaller percentages of repeats in each species (∼6% lower), but *B. pensylvanicus* remained the most repetitive of these *Bombus* genomes. The exact assignment of these repeats to family varied somewhat from the merged repeat library, with each species having a greater fraction of the genome assigned to Retroelements and a smaller fraction to DNA transposons (Supplementary File 1). Improved curation of hymenopteran repeat families will help improve such classifications in the future and enable more robust comparisons or repetitive DNA content among species.

In conclusion, we have provided a new high-quality chromosome-scale genome assembly for *B. pensylvanicus*, a North American bumble bee of conservation concern. Most features of the genome for *B. pensylvanicus* agree with other recent *Bombus* assemblies, including gene numbers, BUSCO completeness, and chromosome synteny. *B. pensylvanicus* was inferred to have an additional chromosome compared with other North American species sequenced to date and did have greater amounts of repetitive DNA than several other species with comparable recent assemblies. However, other bee taxa have similar or greater levels of repetitive DNA (e.g. *Perdita meconis* has 37.3%, *Tetrapedia diversipes* has 38.7%, *Megachile rotundata* has 43.2%) (Kapheim et al. 2015; Schweizer et al. 2024; Santos et al. 2025). Additional sequencing of species from the North American *Thoracobombus* lineage to which *B. pensylvanicus* belongs will assist in determining if elevated repetitive DNA content are representative of this bumble bee group or might be a result of any methodological or technical differences among assemblies. Similarly, sequencing of other North American *Thoracocombus* species (e.g. *B. fervidus* and *B.* californicus) will be necessary to confirm if the 19 putative chromosomes detected here are common across this lineage. The *Thoracobombus* lineage contains several bumble bee species for which there is evidence of declining populations, including the South American species *B. dahlbomii* and the closely-related *B. fervidus*, which are considered endangered and threatened, respectively, by the IUCN (Hatfield et al. 2015b; Morales et al. 2016). The present genome assembly will support comparative conservation genomics analysis to identify possible genetic differences, including factors like genome structure and repetitive DNA, among lineages and species that have suffered declines vs those that have remained relatively stable. Moreover, the *B. pensylvanicus* assembly adds to the growing resource of publicly available bumble bee reference genomes, including threatened and endangered species from other subgenera, such as *B. affinis* (Koch et al. 2023), and efforts such as the Beenome100 project will generate many more assemblies across the genus that will enable further research into the possible role of genomic structure and variation in pollinator declines.

## Supplementary Material

jkaf181_Supplementary_Data

## Data Availability

PacBio long-read sequencing reads are available at the NCBI Sequence Read Archive (SRA) SRR28229882. Short read Arima Hi-C sequencing reads are available on SRA at SRR32887382. RNA sequencing reads are available on SRA at SRR32887379- SRR32887381. The primary assembly accession on NCBI is JBBAXX000000000 in Bioproject PRJNA1083979. Scripts to assemble the genomes follow those used for assembly of the *Bombus huntii* genome and are available on Ag Data Commons (doi: 10.15482/USDA.ADC/25762431.v1; Sim 2024) and other scripts, EGAPx annotations (GFF/GTF), and data files are on Figshare Project “New reference genome assembly for the declining American Bumble Bee *Bombus penyslvanicus*”, https://figshare.com/projects/New_reference_genome_assembly_for_the_declining_American_Bumble_Bee_Bombus_pensylvanicus/242540. Specific datasets available at: https://doi.org/10.6084/m9.figshare.28661291.v1, https://doi.org/10.6084/m9.figshare.28661564.v1, https://doi.org/10.6084/m9.figshare.28661591.v1, https://doi.org/10.6084/m9.figshare.28661705.v1. Supplemental material available at G3 online.
