# Supplementary figures and images for: New reference genome assembly for the declining American Bumble Bee, Bombus pensylvanicus
Source: G3 (Bethesda). 2025 Aug 11;15(10):jkaf181. doi: 10.1093/g3journal/jkaf181 (PMC12506661; doi:10.1093/g3journal/jkaf181)

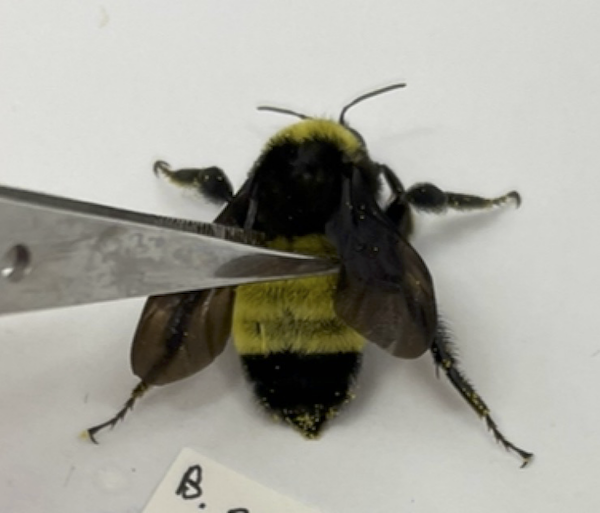

Supplement: jkaf181_Supplementary_Data [file jkaf181_supplementary_data.zip › Supplemental_Figure_S1_G3-2025-406082.tif]

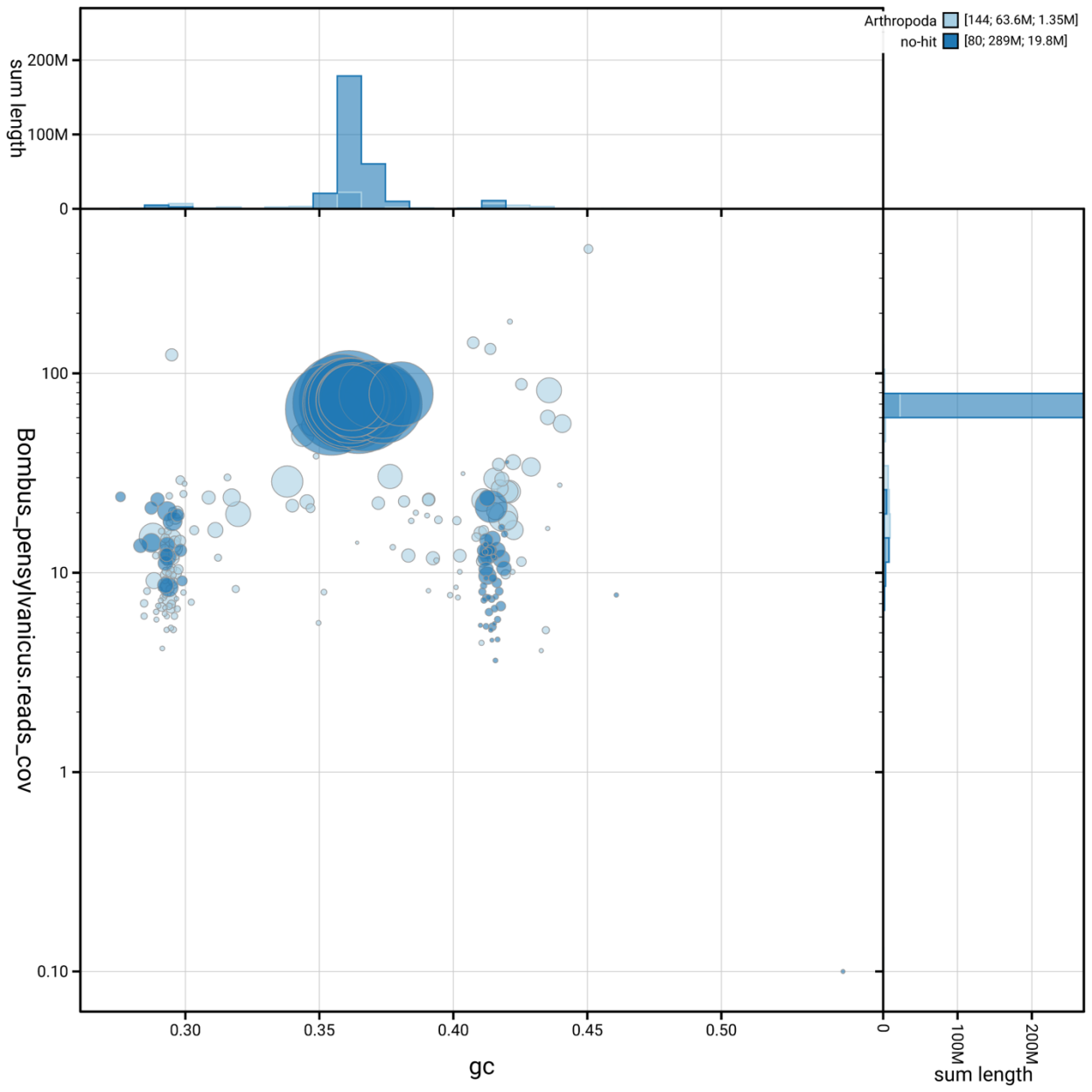

Supplement: jkaf181_Supplementary_Data [file jkaf181_supplementary_data.zip › Supplemental_Figure_S2_G3-2025-406082.pdf]

Density of **genes** and **repeat elements** across 205 non-chromosomal scaffolds

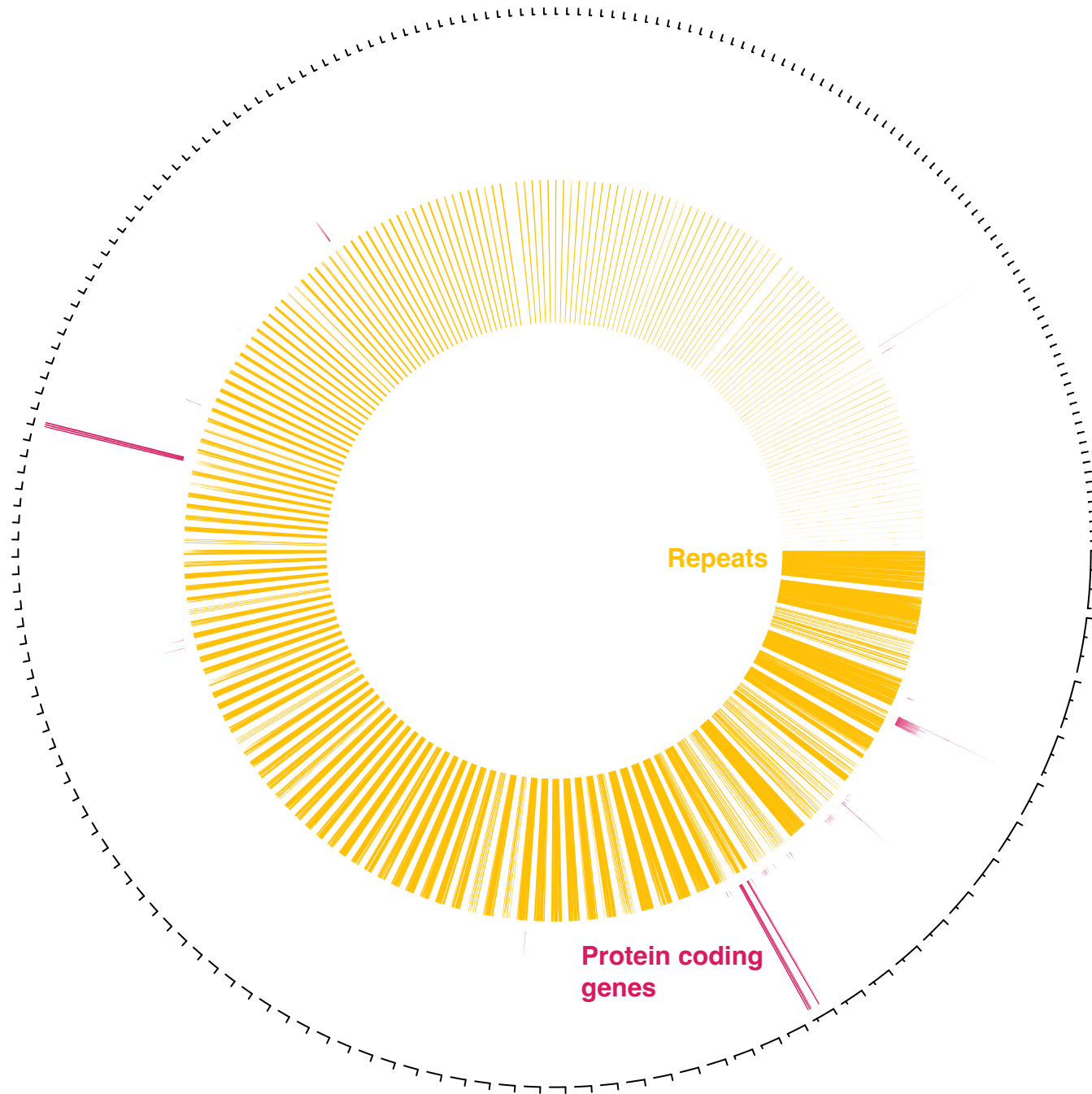

Supplement: jkaf181_Supplementary_Data [file jkaf181_supplementary_data.zip › Supplemental_Figure_S3_G3-2025-406082.pdf]
